# A Deep Learning Approach for Thickness Determination of AZO Thin Films

**DOI:** 10.1021/acsomega.6c10558

**Published:** 2026-10-27

**Authors:** Víctor Manuel Guerrero-Sosa, Angélica Carrillo-Verduzco, Juan Carlos Alonso-Huitrón, Alan Gabriel Amaro-Colin, Miguel Esquivel-García, Xim Bokhimi

**Affiliations:** † Instituto de Investigaciones en Materiales, 7180 Universidad Nacional Autónoma de México, Ciudad de México C.P. 04510, Mexico; ‡ Instituto de Física, Universidad Nacional Autónoma de México, Ciudad de México C.P. 04510, Mexico

## Abstract

We developed a database and a machine learning model to predict the thickness of aluminum-doped zinc oxide (AZO) thin films from their optical transmittance spectra. We measured the spectra of 145 thin films with thicknesses between 100 and 1500 nm and fitted each with a 12-parameter function, one parameter being the film thickness. We divided the thickness range into five regions and modeled the distribution of each fitting parameter in each region as a sum of Gaussian functions. Random sampling from these distributions produced parameter sets used to construct synthetic transmittance spectra (**X**), each paired with its film thickness (**Y**) to form one database sample (**X**, **Y**). We generated 240,000 samples per region, yielding a database of 1.2 million samples for supervised training with thickness as the target. The initial machine learning model, a fully connected neural network, showed poor predictive accuracy. We therefore improved the architecture by incorporating an autoencoder with skip connections to extract the most informative features from the transmittance spectra. This change substantially improved performance, yielding mean absolute errors of 15.8(3) nm on the training set and 15.7­(3) nm on the validation set. When applied to the transmittance spectra of the experimental AZO films, the trained model predicted thicknesses with a mean absolute percentage error of 6.91%. Because the model was trained using data derived from AZO measurements, it applies only to AZO thin films. The methodology, however, is general and can be applied to thin films of any composition.

## Graphical abstract

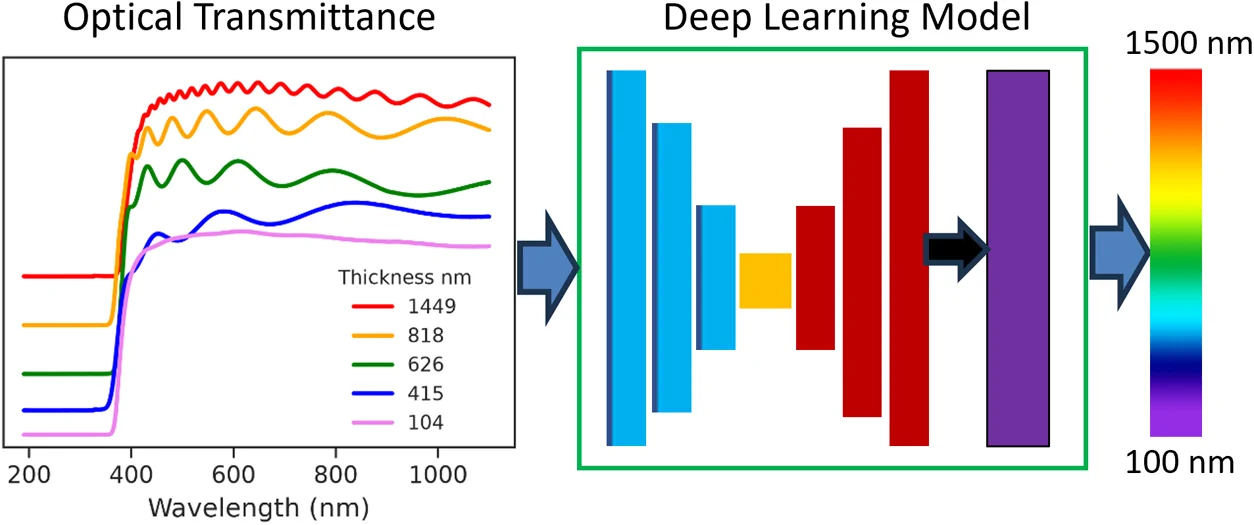

## 1 Introduction

Transparent conductive oxides (TCOs) constitute a class of wide-bandgap semiconductors that combine high optical transparency with remarkable electrical conductivity. These materials are indispensable components in a broad range of optoelectronic technologies, including light-emitting diodes,^1,2^ photovoltaic devices,^3,4^ flat-panel displays,^5,6^ touch screens,^7^ and photodetectors.^8,9^ Among the various TCOs, indium tin oxide remains the most extensively employed in commercial applications. Nevertheless, its widespread use is hindered by the high cost and supply instability of indium. This economic constraint has motivated the search for sustainable and cost-effective alternatives. In this context, aluminum-doped zinc oxide deposited by a variety of techniques has emerged as a promising and economically viable candidate to replace conventional indium tin oxide, offering comparable optoelectronic performance at substantially reduced material cost^10–17^

Thin-film thickness measurements are essential throughout film synthesis, device fabrication, and performance optimization, particularly in semiconductor, optoelectronic, and photonic technologies.^18–20^ Among TCOs, film thickness is one of the most fundamental parameters, as it directly influences both optical transmittance and sheet resistance.

For certain physical deposition techniques, film thickness can be monitored and automatically controlled in situ using a quartz crystal microbalance.^18^ In most cases, however, film thickness is measured using optical methods based on transmittance and/or reflectance spectroscopy. One of the simplest and most widely used approaches for estimating the thickness of transparent, weakly absorbing thin films relies on an analytical formulation that relates film thickness to the refractive index and to wavelengths corresponding to the maxima and minima of the interference fringes in the transmittance and/or reflectance spectra.^21–24^ However, large variations in the refractive index due to the relatively higher value of the absorption coefficient at the absorption edge of TCO thin films lead to substantial deviations in thickness values estimated by this method.^24^ This complicates the method, as reliable thickness values require manually applying outlier-rejection tests to the estimated thickness values. More accurate determinations of thickness and optical constants can be obtained by fitting experimental optical spectra with rigorously developed optical models.^24–29^ Nevertheless, these approaches are often computationally demanding and sensitive to initial parameter selection, requiring an efficient optimization strategy to identify the physically meaningful solution.

Furthermore, achieving high precision can be time-consuming, as it requires comparing theoretical and experimental spectra in fine steps. To overcome these limitations, and enable fast, precise thickness measurements, several studies have applied artificial intelligence (AI) models to predict the thickness of transparent SiO_2_ thin films on silicon, training these models on theoretical reflectance spectra generated for predetermined thickness and refractive index.^30,31^ Building on this approach, deep learning algorithms trained on theoretical reflectance spectra that account for light intensity fluctuation, have also been developed to measure the thickness of silicon based SiN_*x*_ and Si_3_N_4_ thin films, among other certified reference materials.^32–34^ More recently, other AI models have been used to predict film thickness in a variety of material systems. Examples include atomic-layer-deposited ZnO thin films on silicon substrates, using in situ spectroscopic ellipsometry data;^35^ SiO_2_ films on silicon substrates, using a microscopy-based technique,^36^ theoretical reflectance-spectra data sets,^37,38^ or spectroscopic reflectometry data;^39^ and chemically deposited CdS, CdSe and MnO_2_ films, using optical interference fringes.^40^

In this work, we propose an AI-based learning (machine learning) model to determine the thickness of aluminum-doped zinc oxide thin films. The model architecture comprises an autoencoder with skip connections coupled to a fully connected neural network, trained on a database of transmittance spectra constructed by starting from a set of 145 experimental transmittance spectra. The learning model provides a rapid, accurate, and nondestructive means of determining film thickness, offering significant potential for its integration into advanced thin-film characterization workflows.

## 2 Experimental Section

### 2.1 Materials and Methods

Aerosol-assisted chemical vapor deposition (AACVD) was used to synthesize the Al-doped ZnO films. The precursor solution contained 0.2 M zinc acetate dihydrate (Zn­(CH_3_COO)_2_·2H_2_O, ACS reagent, ≥98% Sigma–Aldrich) and 3 atom %. aluminum acetylacetonate (Al­(C_5_H_7_O_2_)_3_, 98%, Sigma–Aldrich), dissolved in a mixture of methanol (ACS reagent, ≥99.8%, Sigma–Aldrich), deionized water (18.2 MΩ-cm, Milli-Q, Millipore), and glacial acetic acid (ACS Reagent, ≥99.7% aldehyde-free, J.T. Baker) in a volume ratio of 87:10:3, respectively. The precursor solution was placed in a spherical glass flask resting in a water-filled beaker, and an ultrasonic nebulizer operating at 1.7 MHz, immersed in the water at the bottom of the beaker, generated the aerosol. Compressed air carried the aerosol to the deposition through a tubular nozzle, which a CNC system moved back and forth over the heated glass substrate (2.5 cm × 2.5 cm × 1 mm). The substrate temperature was set to 410 °C, and films of different thicknesses were obtained by varying the deposition time.

The transmittance spectra were recorded with a PerkinElmer Lambda 35 double-beam UV–vis spectrometer, using air as the reference. Each AZO film deposited on its glass substrate was placed in the sample beam, while the reference beam path was left empty (no beam on bare glass). Spectra were acquired from 190 to 1100 nm in 1 nm steps at a scan speed of 480 nm/min

### 2.2 Generation of the Synthetic Transmission Spectra Database

Applying artificial intelligence to a topic in chemistry or physics requires two kinds of expertise: one on AI methodology and one in the chemistry or physics of the system under study.

In the present work, the goal is to determine the thickness of AZO thin films. Experts in this field must therefore propose the variables that characterize a film, which, together with its thickness, are used to generate a database from which the AI model learns to infer thickness. As specialists in the synthesis and characterization of AZO thin films, we propose the transmittance spectrum, over the wavelength range 190–1100 nm, as the set of variables (**X)** characterizing each film.

The machine learning (ML) model used to infer film thickness is based on supervised learning, with film thickness as the target variable (**Y)**. Each transmittance spectrum comprises 911 values, which serve as the input features of the model.

Training and testing this model require a database with a large number of samples, each having the features and target variables (**X**, **Y**). Building such a database using only experimental transmittance spectra would require a prohibitively large number of experiments, making this approach method impractical. We therefore propose an alternative method for constructing the database: modeling a large number of synthetic samples from a small number of experimental transmittance spectra.

To this end, we prepared 145 thin films with thicknesses ranging from 100 to 1500 nm. For each film, the transmittance spectrum was measured over the wavelength range of 190–1100 nm with a step of 1 nm, generating 911 spectra values; Figure S1 shows a representative spectrum.

Each experimental spectrum was then modeled using the theory of Alonso et al.^22,24^ In this theory, the transmittance spectrum depends on the following parameters (eq 1): light wavelength (λ), film thickness (*d*), absorption coefficient (α), film refractive index (*n*), surface roughness at the film–air interface (σ_1_), surface roughness at the film–substrate interface (σ_2_), carrier concentration (*n*_e_), and substrate refractive index (*n*_g_).

1 $$T=T\left(\lambda ,d,\alpha ,n,\sigma_{1},\sigma_{2},n_{e},n_{g}\right)$$

The absorption coefficient α varies with wavelength λ according to eq 2.

2 $$\alpha \left(\lambda \right)=\alpha_{0}e^{(\left(1240\cdot \beta \left(1/\lambda ‐1/\lambda g\right)\right)}$$

The film refractive index *n* depends on λ according to eq 3

$$n\left(\lambda \right)=\surd \left(A+B\lambda^{2}/\left(\lambda^{2}-C\right)+D\lambda^{2}/\left(\lambda^{2}-E^{2}\right)\right)$$ 3

Substituting eqs ([Disp-formula eq2]) and (3) into eq ([Disp-formula eq1]) expresses the transmittance in terms of the parameters α_*o*_, β, λ_*g*_ (associated with the absorption coefficient); *A*, *B*, *C*, *D*, and *E* (associated with the refractive index); and σ_1_, σ_2_, *n*_e_, and *n*_g_. Together with the film thickness *d*, these 13 parameters fully determine the film transmittance, as shown in eq 4

4 $$T=T\left(\lambda ,d,\alpha_{o},ß,\lambda_{g},A,B,C,D,E,\sigma_{1},\sigma_{2},n_{e},n_{g}\right)$$

Since all films were deposited on the same type of glass substrate, the substrate refractive index (*n*_g_) was the same in every case, ranging between 1.5 to 1.7 with wavelength. The *n*_g_ values as a function of wavelength were obtained from the measured substrate transmittance (*T*_s_) using eq 5.^25^


5 $$n_{g}=1/T_{s}+\surd (1/\left(T_{s}^{2}-1\right)$$

Each experimental transmittance spectrum was fitted to the function in eq 4 by optimizing its 12 free parameters using the Differential Evolution algorithm.^32^ The initial film thickness was estimated either from the maxima and minima of the interference fringes in the spectrum^21^ or, once a trained AI model was available, from the model’s prediction. The initial values of the other 11 parameters were taken from those reported by Alonso et al.^25^ Figure S1 shows a fitted spectrum, and Figure S2 presents the root mean squared error for all fitted spectra. The same procedure was used to determine the thickness of the thinnest films, even though their spectra lack visible interference fringes in the UV.

After fitting the spectra of the 145 experimental films, the resulting thicknesses were grouped into five regions. Region 1 (100–250 nm), Region 2 (250–600 nm), Region 3 (600–950 nm), Region 4 (950–1100 nm), and Region 5 (1100–1500 nm).

Within each thickness region, the distribution of each parameter—*d*, α_o_, *ß*, λ_g_, *A*, *B*, *C*, *D*, *E*, σ_1_, σ_2_, and *n*_e_—was modeled with a Gaussian Mixture Model (GMM). The mean values of these distributions for each region are given in Table 1 and their standard deviation values are given in Table S1.

**Table 1:** Mean Values of the Distribution for Each Variable Used to Calculate the Transmittance

Variable	Region 1	Region 2	Region 3	Region 4	Region 5
d (nm)	1.679 × 10^2^	4.472 × 10^2^	7.993 × 10^2^	1.023 × 10^3^	1.254 × 10^3^
σ_1_(nm)	2.222 ×10^1^	2.333 × 10^1^	2.386 × 10^1^	2.159 × 10^1^	2.235 × 10^1^
σ_2_(nm)	1.601 × 10^1^	2.758 × 10^1^	2.251 × 10^1^	2.000 × 10^1^	2.305 × 10^1^
A	3.748 × 10^0^	3.616 × 10^0^	3.383 × 10^0^	3.280 × 10^0^	3.290 × 10^0^
B	5.189 × 10^5^	9.261 × 10^5^	1.511 × 10^6^	1.546 × 10^6^	1.697 × 10^6^
C(nm)	8.828 × 10^6^	7.356 × 10^6^	3.191 × 10^6^	3.581 × 10^6^	2.232 × 10^6^
D	1.128 × 10^0^	9.053 × 10^–1^	7.166 × 10^–1^	6.186 × 10^–1^	5.995 × 10^–1^
E(nm)	1.890 × 10^2^	2.841 × 10^2^	3.061 × 10^2^	2.850 × 10^2^	3.103 × 10^2^
α_0_(nm^–1^)	6.793 × 10^–3^	8.710 × 10^–3^	6.111 × 10^–3^	6.784 × 10^–3^	7.521 × 10^–3^
β(eV^–1^)	8.261 × 10^0^	1.017 × 10^1^	1.035 × 10^1^	9.196 × 10^0^	9.752 × 10^0^
λ_*g*_(nm)	3.717 × 10^2^	3.589 × 10^2^	3.603 × 10^2^	3.597 × 10^2^	3.547 × 10^2^
*n*_*e*_(m^–3^)	1.913 × 10^26^	2.474 × 10^26^	1.516 × 10^6^	1.400 × 10^26^	1.167 × 10^26^

For each thickness region, we constructed an (**X**, **Y**) sample by drawing random values of the 12 parameters from their respective distributions and using them to compute the transmittance spectrum using eq 4. This process was repeated to obtain 240,000 samples per region. With five thickness regions, the procedure generated 1.2 million synthetic samples in total, each with the transmittance spectrum as the features (**X**) and the corresponding thickness as the target (**Y**).

The database records were randomly shuffled, after which 80% were allocated for training, 10% for validation of the learning model, and the remaining 10% for testing. This shuffling step is critical in the present case, since the data were generated separately for each thickness region.

### 2.3 Infrastructure to Model Construction and Training

The learning model was implemented using Keras 3.11.3,^41^ with TensorFlow 2.20.0 as the backend. Model training and inference were performed on a server with 80 GB Tesla A100 GPUs.

## 3 Results and Discussion

### 3.1 Modeling the Learning System

To construct the learning system’s model, we began with an architecture based on a fully connected neural network, using the transmittance values as input features (Figure 1).

**Figure 1:**
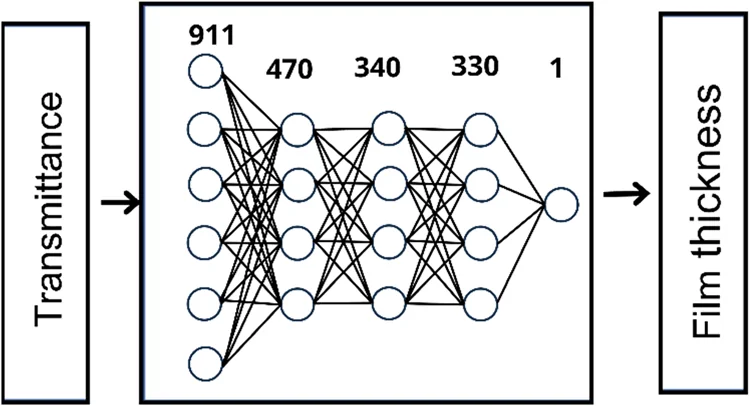
Architecture of the initial learning model developed to predict film thickness.

The trained model was then used to infer thickness values for the testing data. As shown in Figure 2, these predicted values deviate substantially from the true thickness values. This discrepancy is likely due, in part, to the shallow architecture of the model,^42,43^ as well as to the fact that the samples were modeled across five different regions, each governed by its own set of parameters.

**Figure 2:**
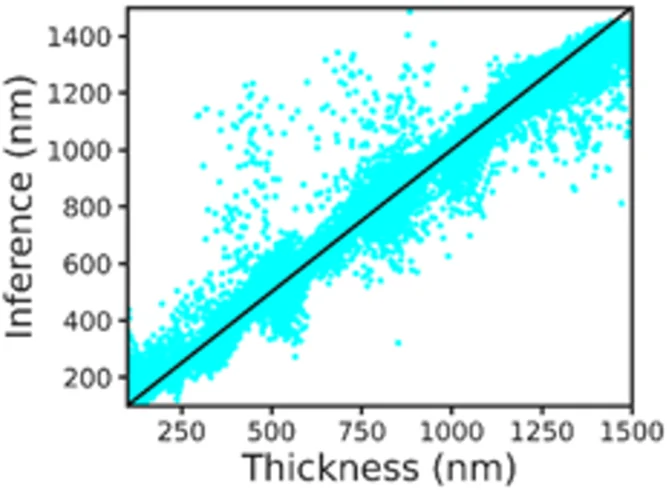
Comparison between predicted and actual film thickness for the test data.

Shallow architectures can be improved by adding an autoencoder^42,44,45^ prior to the fully connected layers, as it enhances the quality of the extracted features. This methodology has been used frequently, yielding superior performance for digit and visual object recognition^46^ and biomedical image segmentation,^47,48^ as well as in the analysis of one-dimensional signals: for example, in the communications field,^49,50^ in damage diagnosis in Carbon Fiber Reinforced Polymer plates using Lamb waves,^51^ in the analysis of the trajectory of secretory vesicle dynamics,^52^ and in the analysis of electrocardiograms.^53^ Autoencoders can further improve their output by incorporating skip connections between the encoder and decoder.^47,54^

Following this precedent, we added an autoencoder with skip connections to the initial architecture of our leaning model, constructing a deep learning model. The autoencoder was built with convolutional layers for the encoder and deconvolutional layers for the decoder. This new deep learning model extracts richer features from the transmittance spectrum, improving learning and producing better predictions of film thickness.

### 3.2 Deep Learning Model Architecture

The final network architecture consists of two main components (Figure 3). The first is an autoencoder with short skip connections between corresponding layers, similar to the U-Net architecture.^55^ The second component is a fully connected multilayer neural network.

**Figure 3:**
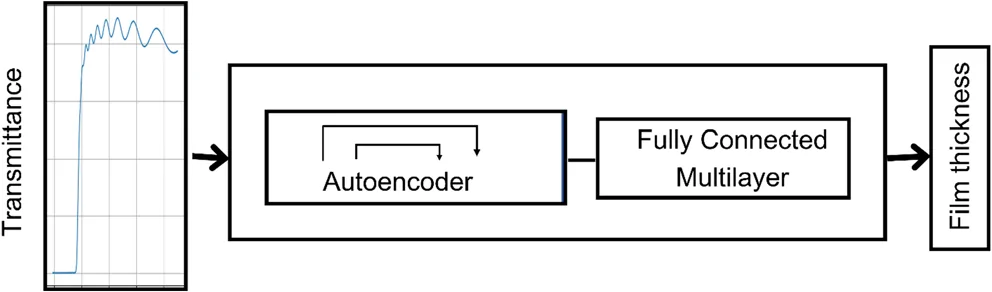
Deep learning model developed for the prediction of film thickness.

The skip connections in the autoencoder perform an identity mapping, transferring the output of each convolutional block in the encoder to the input of the corresponding deconvolutional block in the decoder. As in ResNet^55^ and other multilayer architectures of deep learning models,^56^ these short connections facilitate the learning by mitigating the degradation of gradients caused by the network’s depth^55^ or by strong nonlinearities in the problem.^56^

In the following paragraphs and figures, the number of the feature maps associated with each layer corresponds to the optimized configuration after tuning the hyperparameters of the deep learning architecture.

The encoder architecture has four blocks (Figure 4). The first one includes two convolutional layers with a kernel size of 150. Batch normalization is applied after the second and third layers to enhance converge speed, training stability, and regularization.^57^ The second and third convolutional layers used Leaky ReLU activation functions, and generated 21 feature maps, each containing 911 features. The block output serves as the input for the second encoder block and as a skip connection—through concatenation—to the last decoder block. This connection assists the decoder in localizing high-resolution features from the encoder.^47^

**Figure 4:**
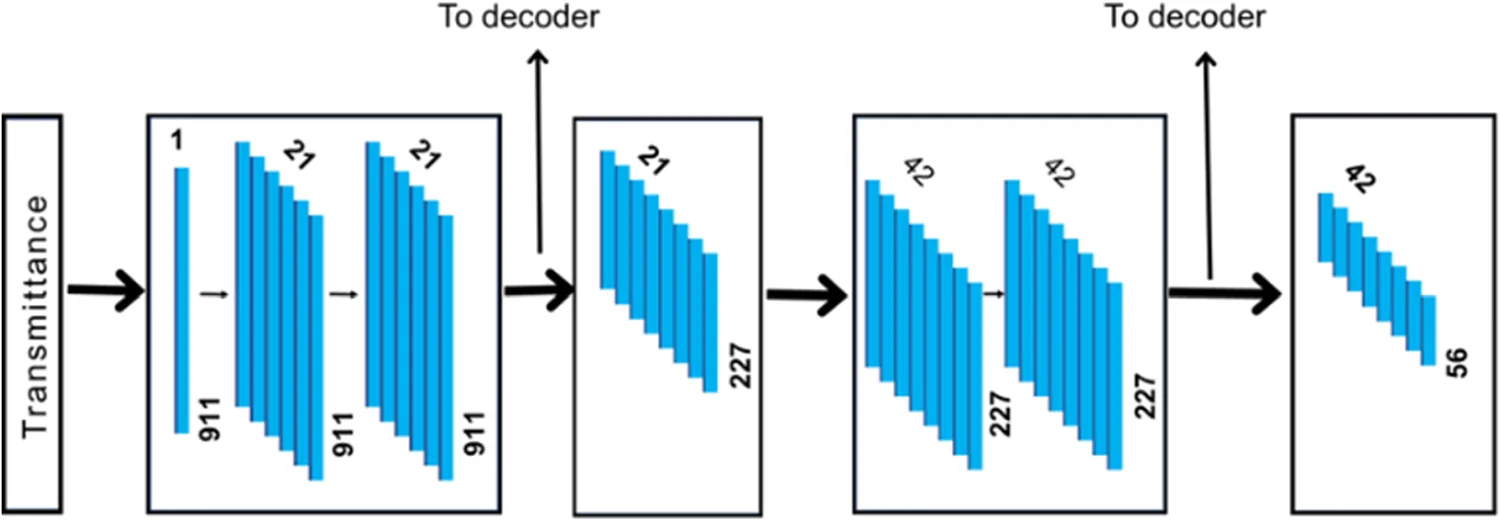
Encoder blocks showing the details of their layer composition.

The second encoder block contains a single layer (Figure 4), implemented as an average pooling operation with a pool size and stride of 4. This layer produces an output composed of 21 feature maps, each with 227 features.

The encoder’s third block consists of two convolutional layers with a kernel size of 50 (Figure 4). Each layer includes batch normalization and uses the Leaky ReLU activation function, and has 42 feature maps, each containing 227 features. The output of this block serves as the input for the fourth encoder block and is also concatenated as a short skip connection to the second block of the decoder. This skip connection helps the decoder recover high-resolution spatial features from the encoder.

The fourth and final encoder block contains a single layer (Figure 4), implemented as an average pooling operation with a pool size and stride of 4. This layer generates 42 feature maps, each with 56 features. The output of this block is fed into the module that constructs the latent space, a key component in autoencoder architectures.

The latent space module (Figure 5) comprises two convolutional layers with a kernel size of 5. Each layer incorporates batch normalization and the Leaky ReLU activation function, and produces 84 feature maps, each containing 56 features. The output of this module serves as the input to the first block of the decoder.

**Figure 5:**
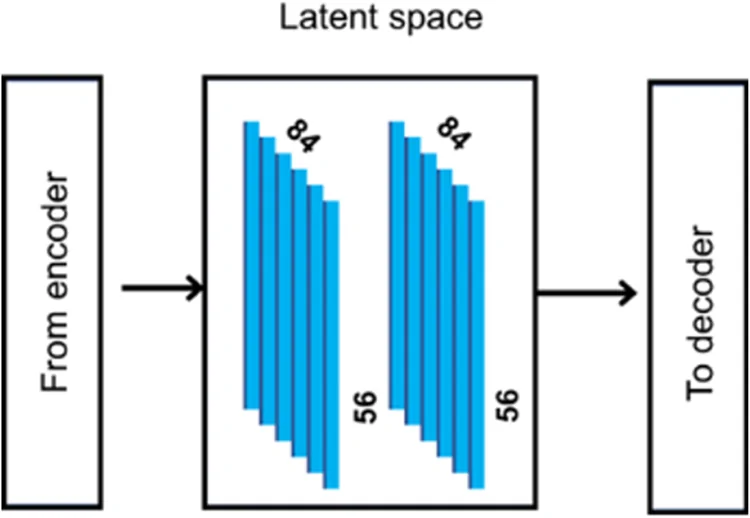
Generating the latent space.

The first block of the decoder (Figure 6) consists of a single layer that up-samples its input, which originates from the latent space block, using the nearest-neighbor algorithm with a scale factor of 4. In this process, each input feature value is sequentially repeated four times, producing an output layer with 84 feature maps. After zero padding, each feature map contains 227 features.

**Figure 6:**
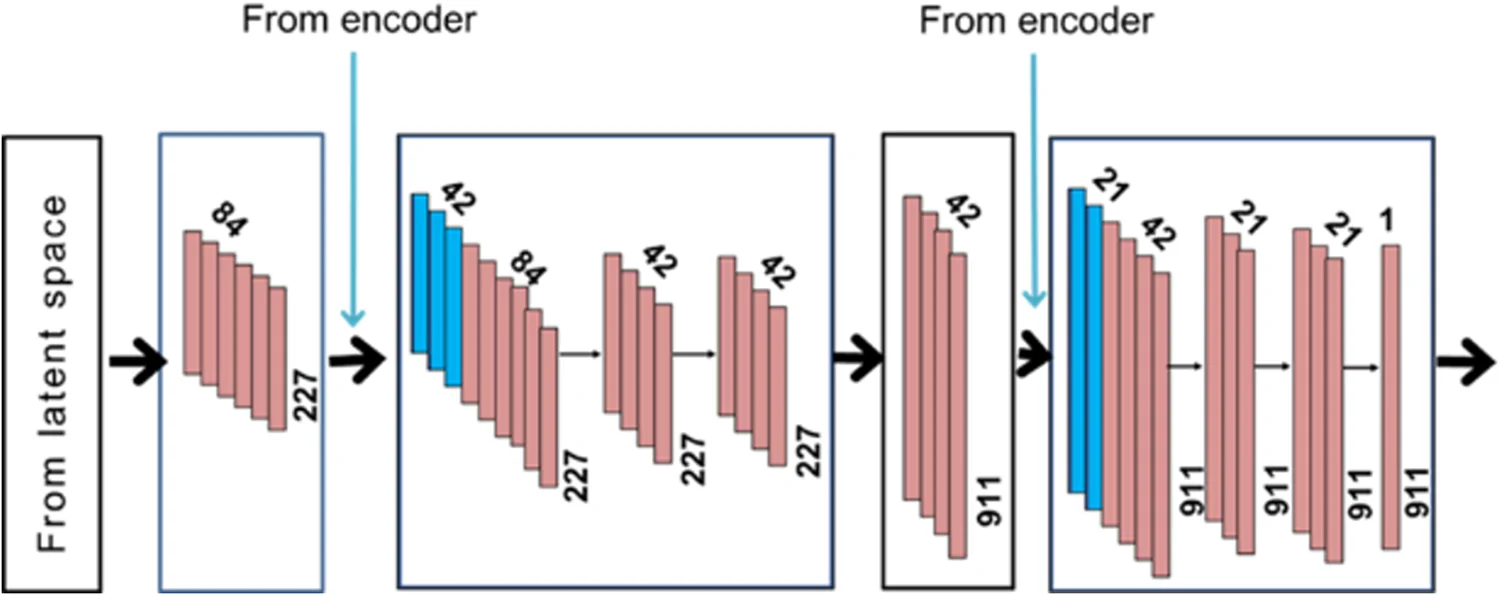
Decoder blocks showing details of their layer composition.

The second block of the decoder comprises three layers. The first one is formed by concatenating the 42 feature maps from the third encoder block with the 84 feature maps from the first decoder block. This concatenation produces 126 feature maps, each containing 227 features. The subsequent two layers are convolutional, each with a kernel size of 50, batch normalization and the Leaky RELU activation function. Both layers generate 42 feature maps, each consisting of 227 features. The output of this block serves as the input for the third decoder block.

This third decoder block contains a single layer that performs up-sampling using the nearest-neighbor algorithm with a scale factor of 4, each input feature value is repeated sequentially four times, generating an output layer with 42 feature maps each. After zero padding, each feature map contains 911 features. The fourth block of the decoder consists of four layers. The first layer concatenates the 21 feature maps from the first encoder block with the 42 feature maps from the third decoder block, resulting in 63 feature maps, each having 911 features. This layer is followed by two convolutional layers, each with a kernel size of 150, batch normalization and the Leaky ReLU activation function. Both layers produce 21 feature maps with 911 features each. The final layer is a convolutional layer with a kernel size of 1, no batch normalization and the Leaky ReLU activation function. It outputs a single feature map with 911 features. In this final layer, the filter effectively learns a weighted combination of the 21 input feature maps to produce the single output feature map. The output of this block serves as the input of the fully connected neural network.

The output tensor of the last decoder block, with dimensions (911, 1), is reshaped into a vector containing 911 elements that constitute the input features of the fully connected neural network.

The fully connected neural network comprises three hidden layers (Figure 1). The first hidden layer contains 470 nodes, followed with a dropout rate of 0.35 and batch normalization. The second hidden layer includes 340 nodes, a dropout rate of 0.35, and batch normalization. The third hidden layer has 330 nodes also followed by dropout (0.35) and batch normalization. The output layer consists of a single node with the ReLU activation function.

### 3.3 Training the Model

As mentioned above, from the total data set of 1.2 million samples, 80% were used to train the model, 10% for validation at each epoch, and the remaining 10% for testing after training was completed. The model was trained for 1000 epochs using mean absolute error (MAE) as the loss function and Adam as the optimizer, with a cosine-decreasing learning rate starting at an initial value of 0.0001 and a batch size of 512, resulting in 2019 weight updates per epoch. The final training and validation loss errors were 15.8(3) nm and 15.7(3) nm, respectively (Figure 7), with the corresponding mean absolute percentage errors (MAPE) of 5.3(2)% and 5.3(2)%, demonstrating the high accuracy of the model.

**Figure 7:**
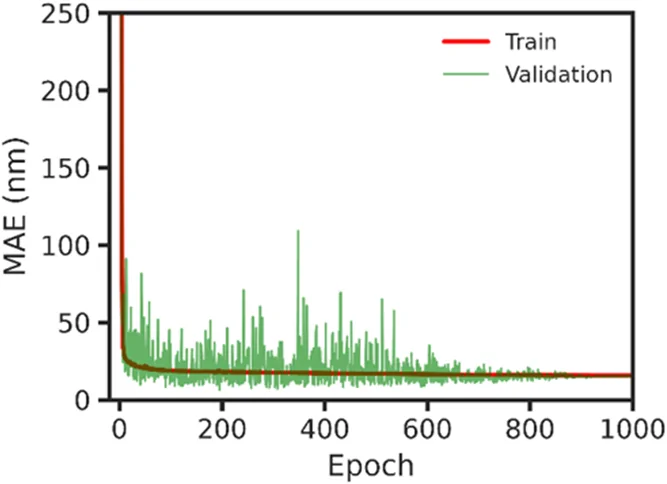
Loss function value over training epochs.

Figure 8 shows the precision of the model’s predicted thickness for the 120,000 samples in the test set, highlighting the excellent performance of the deep learning model. This figure reveals a marked difference compared to the predicted thickness obtained with the fully connected neural network (Figure 2).

**Figure 8:**
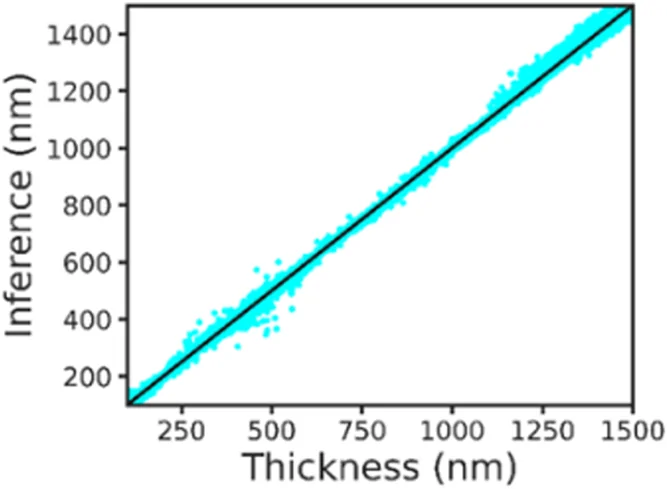
Comparison between predicted and actual film thickness for the test data.

### 3.4 Evaluation of the Model with the Experimental Films

The predicted thickness values for the 145 experimental thin films—used to generate the samples for the learning system—showed a mean absolute percentage error of 6.91% compared to the thickness obtained from fitting the experimental transmittance spectra. A detailed comparison of the predicted and experimental values across the wavelength range used in the experiments are shown in Figure 9.

**Figure 9:**
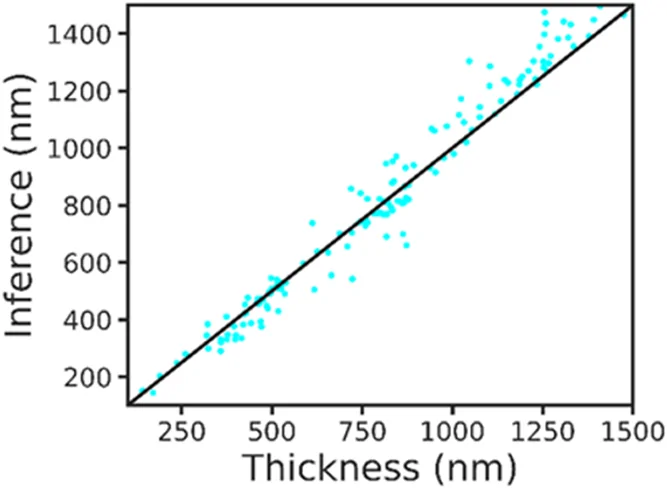
Comparison between predicted and experimental film thicknesses.

## 4 Conclusions

We describe the development of a database and a machine learning model to predict the thickness of aluminum-doped zinc oxide thin films from their optical transmittance spectra. To build the database we prepared and measured the optical transmittance spectra of 145 thin films with thicknesses ranging from 100 to 1500 nm, over the wavelengths range 190 to 1100 nm. We fitted each spectrum using the theory of Alonso et al.^25,27^ In which the transmittance is described by a function of 12 free parameters, one of which is the film thickness.

To generate synthetic data, we divided the thickness range into five regions and, within each region, modeled the distribution of every fitting parameter as a sum of Gaussian functions. We then sampled randomly from these distributions to obtain values for all 12 parameters and used them to construct a synthetic transmittance spectrum with Alonso’s theory. Each spectrum, paired with its film thickness, forms one database sample (**X**, **Y**), where **X** is the transmittance spectrum and **Y** is the thickness. We generated 240,000 samples per region, yielding a database of 1.2 million samples.

We used this database to train machine learning models by supervised learning, with film thickness as the target variable. An initial fully connected neural network showed poor predictive accuracy. We therefore improved the architecture by incorporating an autoencoder with skip connections to extract the most informative features from the transmittance spectra. This change substantially improved performance, yielding mean absolute errors (MAE) of 15.8(3) nm on the training set and 15.7(3) nm on the validations set, with the corresponding mean absolute percentage errors (MAPE) of 5.3(2)% and 5.3(2)%. When applied to the transmittance spectra of the experimental AZO films, the trained model predicted thicknesses with a mean absolute percentage error (MAPE) of 6.91% and a mean absolute error (MAE) error of 22.7(3) nm. Because the model was trained on data derived from AZO measurements, it applies only to AZO thin films. The methodology, however, is general: it can be applied to thin films of any composition, and on any problem in which a material property depends on the film’s transmittance, such as transparent ferromagnets,^58^ band gap,^59^ and surface roughness.^60^

## Supplementary Material



## Notes

Experimental and modeled transmittance spectrum of a film with a thickness of 1003.4 nm (Figures S1); Root Mean Squared Error values as a function of film thickness obtained after fitting the transmittance spectra (Figure S2); and standard deviation values of the distribution for each variable used to calculate the transmittance (Table S1) (PDF)
