# Supplementary material for: A Deep Learning Approach for Thickness Determination of AZO Thin Films
Source: ACS Omega. 2026 Oct 27;11(44):69373–80. doi: 10.1021/acsomega.6c10558 (PMC13756646; doi:10.1021/acsomega.6c10558)
Supplement: Supplementary file 1 [file ao6c10558_si_001.pdf]

Supporting Information (SI) for ACS Applied Materials & Interfases.

A Deep Learning Approach for Thickness Determination of AZO Thin Films

Víctor Manuel Guerrero-Sosa[a], Angélica Carrillo-Verduzco[a], Juan Carlos Alonso-Huitrón[a] Alan Gabriel Amaro-Colin[b], Miguel Esquivel-García[b], and Xim Bokhimi[b,]*

a. Instituto de Investigaciones en Materiales, Universidad Nacional Autónoma de México, Ciudad de México, C.P. 04510, Mexico.

b. Instituto de Física, Universidad Nacional Autónoma de México, Ciudad de México, C.P. 04510, Mexico.

**Email: bokhimi@fisica.unam.mx*

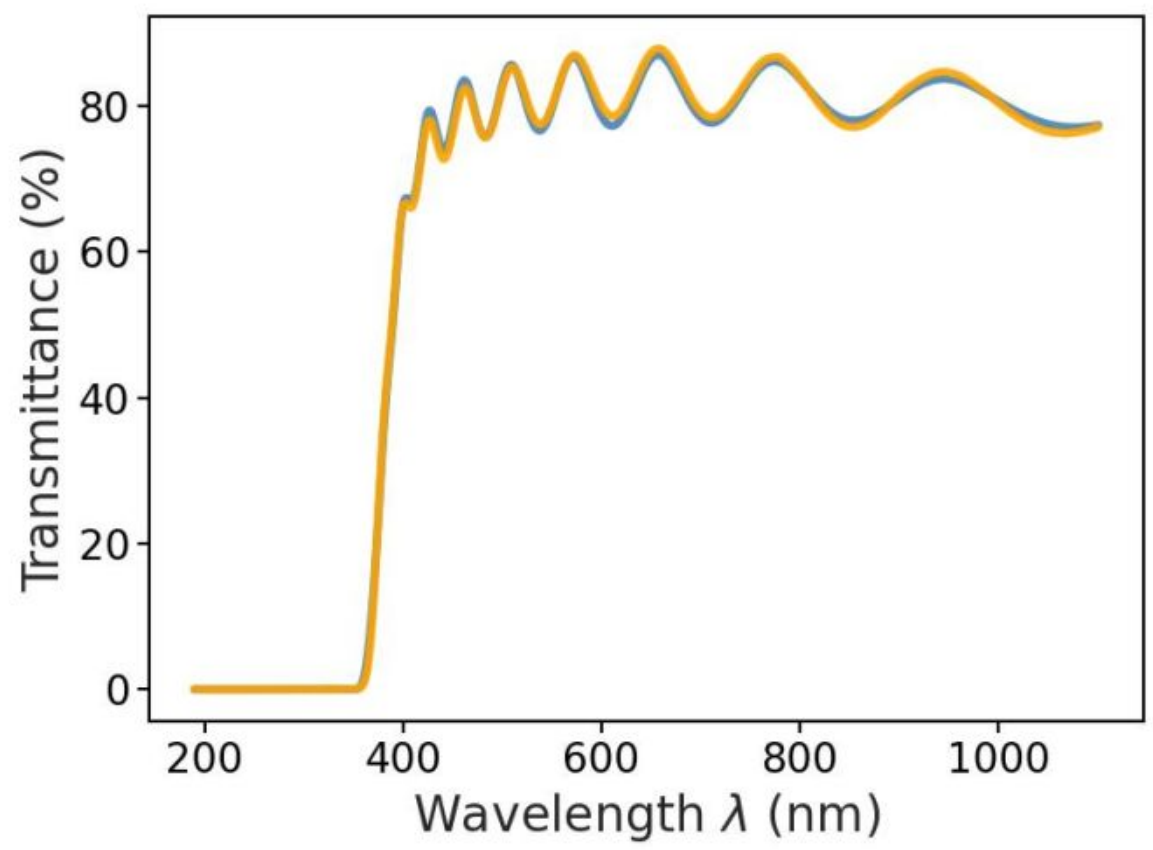

Figure S1. Experimental (blue) and modeled (orange) transmittance spectrum of a film with a thickness of 1003.4 nm.

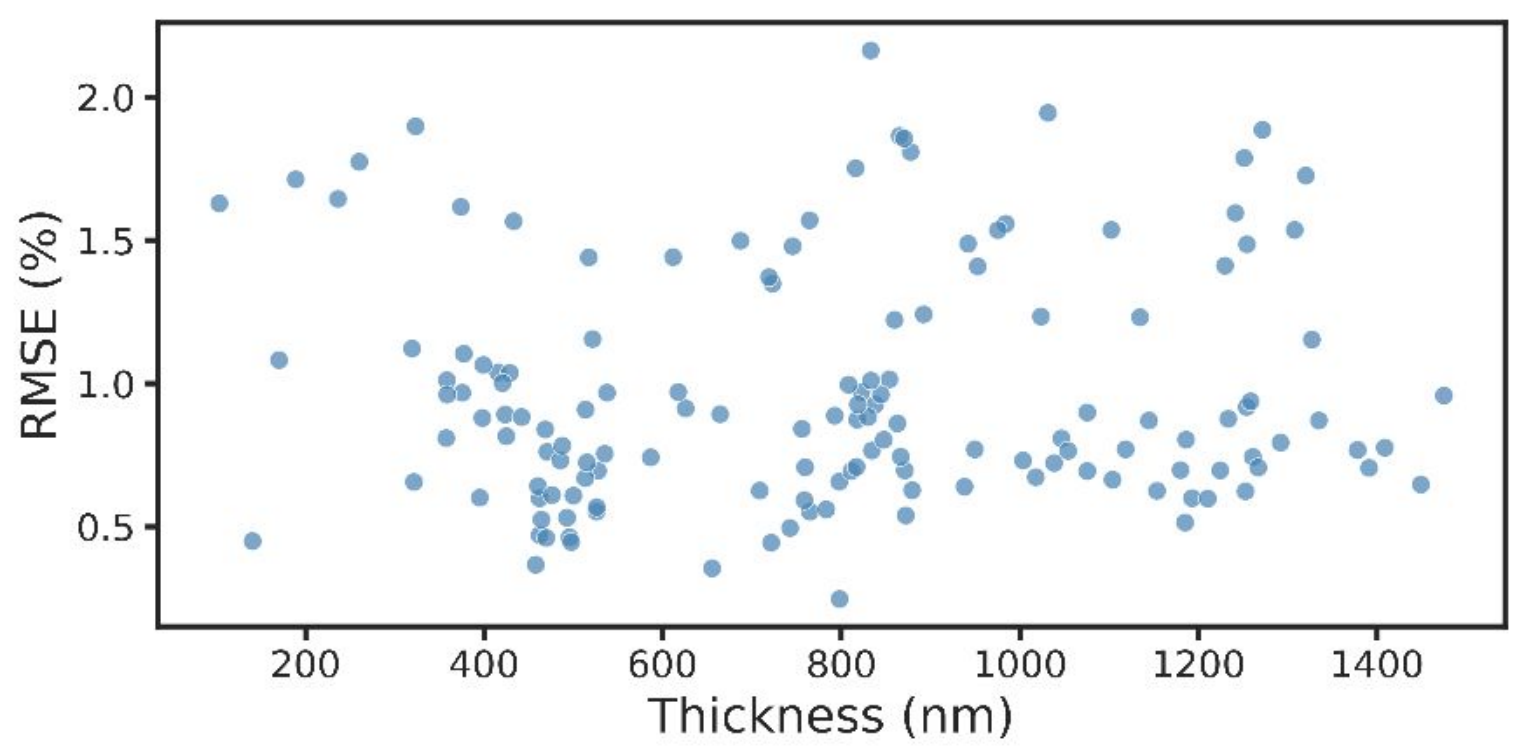

Figure S2. RMSE values as a function of film thickness obtained after fitting the transmittance spectra.

Table S1: Standard deviation values of the distribution for each variable used to calculate the transmittance.

Variable	Region 1	Region 2	Region 3	Region 4	Region 5
d (nm)	4.469e+01	7.021e+01	8.096e+01	3.701e+01	9.360e+01
σ_1(nm)	1.952e+01	1.485e+01	1.551e+01	1.349e+01	8.364e+00
σ_2(nm)	9.307e+00	1.941e+01	1.303e+01	1.152e+01	1.061e+01
A	4.234e-01	4.257e-01	2.860e-01	9.499e-02	1.194e-01
B	4.410e+05	7.236e+05	6.537e+05	4.458e+05	5.102e+05
C(nm)	3.966e+06	5.009e+06	2.720e+06	3.175e+06	7.721e+05
D	1.195e+00	5.781e-01	4.392e-01	1.643e-01	1.131e-01
E(nm)	7.626e+01	6.482e+01	4.604e+01	6.726e+01	3.794e+01
α_0(nm^{-1})	3.758e-03	3.834e-03	3.305e-03	3.508e-03	3.400e-03
β(eV^{-1})	2.521e+00	1.309e+00	1.417e+00	9.025e-01	2.434e+00
λ_g(nm)	1.542e+01	6.887e+00	5.673e+00	1.258e+01	6.930e+00
n_e(m^{-3})	2.792e+26	1.762e+26	1.116e+26	1.014e+26	4.389e+25
